# Impact of Seasonal PM_2.5_ Exposure on Metabolic and Hormonal Profiles in Healthy Individuals and Individuals with Metabolic Syndrome in Chiang Mai, Thailand

**DOI:** 10.3390/toxics13080614

**Published:** 2025-07-23

**Authors:** Sharjeel Shakeel, Shamsa Sabir, Wason Parklak, Sawaeng Kawichai, Praporn Kijkuokool, Wiritphon Khiaolaongam, Pakaphorn Ngamsang, Putita Jiraya, Hataichanok Chuljerm, Puriwat Fakfum, Kanokwan Kulprachakarn

**Affiliations:** 1 Environmental Science Research Center, Faculty of Science, Chiang Mai University, Chiang Mai 50200, Thailand; sheerikhan995@gmail.com; 2 School of Health Sciences Research, Research Institute for Health Sciences, Chiang Mai University, Chiang Mai 50200, Thailand; shamsasabir01@gmail.com (S.S.); praporn_k@cmu.ac.th (P.K.); wiritphon_k@cmu.ac.th (W.K.); pakaporn_ng@cmu.ac.th (P.N.); putita_jiraya@cmu.ac.th (P.J.); hataichanok.ch@cmu.ac.th (H.C.); puriwat_f@cmu.ac.th (P.F.); 3 Research Center for Non-Infectious Diseases and Environmental Health, Research Institute for Health Sciences, Chiang Mai University, Chiang Mai 50200, Thailand; wason.p@cmu.ac.th (W.P.); sawaeng.kaw@cmu.ac.th (S.K.); 4 Global Health and Chronic Conditions Research Center, Chiang Mai University, Chiang Mai 50200, Thailand; 5 Department of Family Medicine, Faculty of Medicine, Chiang Mai University, Chiang Mai 50200, Thailand

**Keywords:** PM_2.5_, metabolic syndrome, insulin, leptin, adiponectin, HOMA-IR, air pollution, Chiang Mai

## Abstract

Exposure to fine particulate matter (PM_2.5_) is linked to metabolic dysfunction, yet evidence on its impact on hormonal regulation remains limited. This study examined seasonal changes in insulin, adiponectin, leptin, and HOMA-IR levels among healthy individuals and those with metabolic syndrome (MS) in Chiang Mai, Thailand. Fifty participants (25 healthy, 25 with MS) were assessed during high (February–April)- and low (May–July)-PM_2.5_ seasons. Insulin levels increased in healthy individuals (mean: 9.3 to 14.9 µIU/mL; *p* = 0.051) and decreased in participants with MS (22.0 to 13.7 µIU/mL; *p* = 0.214), with a significant interaction effect (*p* = 0.020). Leptin increased significantly in both groups, but more markedly in the MS group (*p* < 0.001), also with a significant interaction (*p* < 0.001). HOMA-IR rose significantly in healthy individuals (*p* = 0.036) but not in participants with MS. Adiponectin remained stable across groups and seasons. At baseline, the MS group had significantly higher rates of diabetes (*p* = 0.050), hypertension (*p* = 0.001), and hyperlipidemia (*p* = 0.049). These findings suggest that PM_2.5_ may influence metabolic and hormonal profiles, particularly in individuals with existing metabolic disorders.

## 1. Introduction

Fine particulate matter (PM_2.5_), defined as particles with a diameter of less than 2.5 micrometers, represents a major global public health concern, particularly in urban and industrialized regions [1]. PM_2.5_ originates from diverse sources such as motor vehicles, industrial activities, and biomass burning. Its small size allows it to penetrate deep into the respiratory tract and enter the systemic circulation [2], contributing to a wide range of adverse health outcomes, including respiratory, cardiovascular, and metabolic diseases [3]. The physicochemical properties of PM_2.5_ enable it to remain suspended in the air for extended periods, increasing the duration and intensity of human exposure, especially in highly polluted areas [4,5]. Numerous studies have demonstrated that exposure to PM_2.5_ can induce oxidative stress and systemic inflammation, mechanisms that are closely linked to the development of metabolic disturbances such as insulin resistance, obesity, and type 2 diabetes [6,7]. Furthermore, long-term exposure to PM_2.5_ has been associated with alterations in lipid metabolism [8] and disruption of hormonal pathways, particularly those involving the hypothalamic–pituitary–adrenal (HPA) axis and insulin signaling [9]. These hormonal imbalances can exacerbate metabolic dysfunction, contributing to the pathogenesis of chronic diseases such as metabolic syndrome and diabetes [3,5]. PM_2.5_ can contribute to the pathogenesis of metabolic syndrome through several biological pathways. One key mechanism is oxidative stress, where inhaled particles generate reactive oxygen species (ROS), leading to cellular damage and inflammation in metabolic tissues such as adipose, liver, and muscle tissues [10]. This oxidative stress can trigger insulin resistance and lipid abnormalities. PM_2.5_ also promotes adipose tissue inflammation by activating macrophages and increasing the secretion of pro-inflammatory cytokines like TNF-α and IL-6, which are known to impair insulin signaling [11]. Furthermore, long-term PM_2.5_ exposure may disrupt neuroendocrine regulation via the hypothalamic–pituitary–adrenal (HPA) axis, contributing to hormonal imbalances and metabolic dysregulation [12]. These mechanisms are supported by both epidemiological and experimental studies, reinforcing the role of PM_2.5_ as a metabolic disruptor.

Metabolic syndrome (MS) is a cluster of interrelated conditions, including abdominal obesity, hypertension, hyperglycemia, dyslipidemia, and insulin resistance, that significantly increase the risk of cardiovascular disease and type 2 diabetes [13]. The growing prevalence of MS worldwide has been closely linked not only to lifestyle factors but also to environmental exposures, including air pollution [14,15]. Chronic exposure to PM_2.5_ may play a role in exacerbating insulin resistance and abdominal fat deposition, which are central features of metabolic syndrome. Several hormones are implicated in the development and progression of metabolic syndrome. Insulin is a key regulator of glucose metabolism, and resistance to its action is a hallmark of MS [16]. Leptin, an adipocyte-derived hormone, plays a role in appetite regulation and energy expenditure but is often elevated in individuals with obesity, leading to leptin resistance [17]. In contrast, adiponectin, another adipokine, has anti-inflammatory and insulin-sensitizing properties and is typically reduced in individuals with MS. The Homeostatic Model Assessment of Insulin Resistance (HOMA-IR) is a validated index that is used to estimate insulin resistance based on fasting glucose and insulin levels [18]. Alterations in these hormonal markers reflect the underlying metabolic dysregulation associated with both obesity and environmental exposures such as PM_2.5_ [19].

In Chiang Mai, Thailand, seasonal agricultural burning activities significantly elevate PM_2.5_ levels, particularly during the dry season from February to April. This distinct pattern of seasonal variation offers a unique opportunity to study the health impacts of short-term and long-term PM_2.5_ exposure [20]. Understanding the link between environmental pollution and metabolic dysfunction in such settings is essential for developing targeted public health interventions.

**Figure 1:**
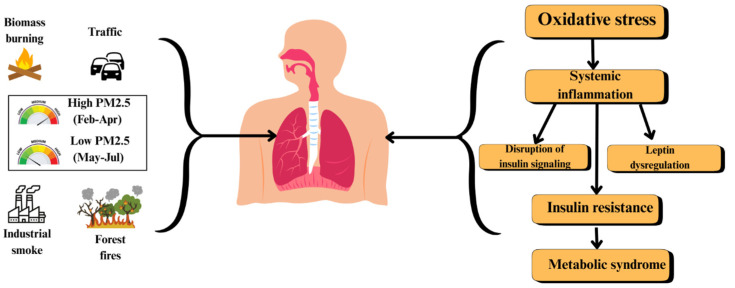
Pathway linking PM_2.5_ exposure to metabolic syndrome.

Previous research has linked PM_2.5_ exposure to metabolic abnormalities, including insulin resistance, dyslipidemia, and systemic inflammation, which together contribute to cardiovascular and endocrine diseases [7,9]. Oxidative stress plays a central role in these processes, triggering chronic inflammation and impairing normal cellular functions [4,21]. Additionally, PM_2.5_-induced hormonal disruptions, such as altered insulin signaling and adipokine imbalance, have been implicated in the worsening of metabolic disorders [14], as demonstrated in Figure 1.

Given the high levels of air pollution in Chiang Mai, the local population may face an elevated risk of metabolic and hormonal imbalances associated with PM_2.5_ exposure [22]. It is crucial to comprehensively evaluate these impacts to inform preventive health strategies and reduce the burden of pollution-related metabolic diseases.

Therefore, this study aims to assess the effects of PM_2.5_ exposure on metabolic and hormonal parameters, specifically insulin, adiponectin, leptin, and HOMA-IR, by comparing healthy individuals and individuals with metabolic syndrome across two distinct PM_2.5_ seasons in Chiang Mai, Thailand. The findings are intended to contribute to the growing body of evidence linking air pollution to endocrine and metabolic health, with the goal of supporting effective public health policies and interventions.

## 2. Materials and Methods

### 2.1. Air Quality Monitoring

**Figure 2:**
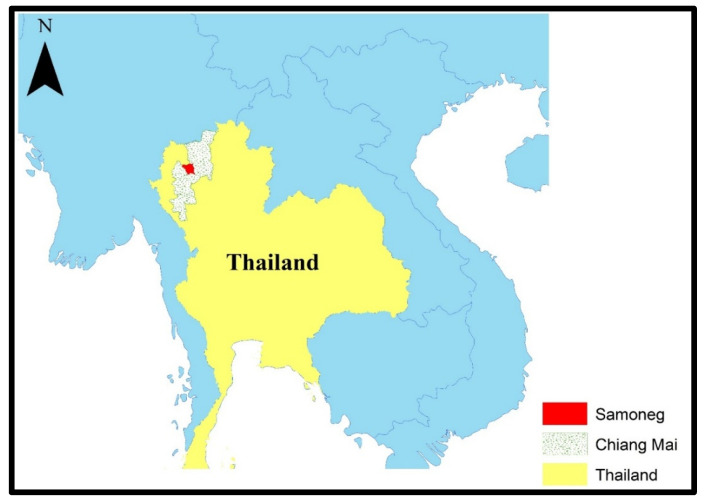
Map showing study area.

Ambient air quality and PM_2.5_ concentrations were monitored in Samoeng District, Chiang Mai Province, as shown in Figure 2. Air quality sensors were installed by the Subdistrict Administrative Organizations and continuously recorded PM_2.5_ levels. Data were obtained from the Northern Thailand Air Quality Health Index (NTAQHI) system, operated by the Research Institute for Health Sciences (RIHES), Chiang Mai University, Chiang Mai, Thailand. Two distinct exposure periods were defined: the high-PM_2.5_ season (February–April 2023) and the low-PM_2.5_ season (May–July 2023). Sensor calibration, data averaging, and quality assurance followed the standard operating procedures established by RIHES.

### 2.2. Study Participants

Adults aged 25 to 60 years who had resided in Samoeng District, Chiang Mai, for at least five years were recruited. Participants were classified into two groups: healthy individuals and individuals with metabolic syndrome (MS). The recruitment was carried out at Subdistrict Health Promoting Hospitals and the Samoeng District Hospital. Healthy participants were free from chronic diseases such as cancer, cardiovascular diseases, and chronic kidney disease and had no active infections at the time of enrollment. Individuals classified under the MS group met at least three of the following criteria:

- Fasting blood glucose ≥100 mg/dL;
- Waist circumference >90 cm for men or >80 cm for women;
- Blood pressure ≥130/85 mmHg;
- Triglyceride levels ≥150 mg/dL;
- High-density lipoprotein cholesterol (HDL-C) <40 mg/dL for men and <50 mg/dL for women.

The exclusion criteria included pregnancy, recent surgery, active infections, a history of substance abuse, psychiatric or neurological disorders, or lack of compliance with study procedures.

### 2.3. Study Design and Data Collection

This study employed a prospective observational design to evaluate seasonal changes in metabolic and hormonal profiles in relation to PM_2.5_ exposure. Participants attended two study visits: one during the high-PM_2.5_ season and another during the low-PM_2.5_ season.

At each visit, data collection included the following:

- Structured questionnaires capturing demographic, lifestyle, and health information;
- Anthropometric measurements: body weight, height, waist circumference, hip circumference, and blood pressure;
- Blood sample collection for laboratory analysis.

Initially, 53 participants were enrolled (26 healthy and 27 with MS); however, 3 participants were lost to follow-up, resulting in a final sample size of 50 participants (25 per group).

### 2.4. Laboratory Analysis

Fasting blood samples were collected during both visits and processed for the evaluation of metabolic and hormonal parameters.

Clinical chemistry parameters, including fasting glucose and lipid profiles, were analyzed using the VITROS XT 7600 Integrated System (Ortho Clinical Diagnostics, Raritan, NJ, USA).

For hormonal assessments, insulin (catalog number; RAB0327-1KT), adiponectin (catalog number; RAB0005-1KT), and leptin (catalog number; RAB0333-1KT) concentrations were measured using enzyme-linked immunosorbent assay (ELISA) kits. All ELISA kits were obtained from Sigma Aldrich (St. Louis, MO, USA).

Homeostatic Model Assessment for Insulin Resistance (HOMA-IR) was calculated using the following formula:

HOMA-IR = Fasting Insulin (µIU/mL) × Fasting Glucose (mg/dL)/450

Absorbance readings for ELISAs were measured using a VICTOR Nivo multimode microplate reader (Revvity, Waltham, MA, USA). All laboratory procedures followed the manufacturers’ protocols to ensure the precision and reliability of results.

### 2.5. Ethical Considerations

The study protocol was approved by the Human Experimentation Committee of the Research Institute for Health Sciences, Chiang Mai University (Approval No. 03/2023), in accordance with the Declaration of Helsinki. All participants provided written informed consent prior to enrollment.

### 2.6. Statistical Analysis

Statistical analysis was performed using the Stata software version 17.0. Descriptive statistics were used to summarize participants’ characteristics and biomarker levels, including insulin, adiponectin, leptin, and HOMA-IR. Continuous variables were presented as means with standard deviations (SDs), while categorical variables were reported as frequencies and percentages. To examine the effects of metabolic health status (healthy vs. metabolic syndrome), seasonal exposure (high vs. low PM_2.5_), and their interaction on biomarker levels, linear mixed-effects models were applied. Each model included fixed effects for metabolic group, season, and the group × season interaction, with a random intercept for each participant to account for repeated measurements and within-subject variability.

This approach allowed for individual-level heterogeneity in baseline hormone levels and properly accounted for the dependency of longitudinal observations. Model outputs included parameter estimates with 95% confidence intervals (CIs). Statistical significance was set at a two-sided alpha level of 0.05. To visualize model-predicted values, marginal means and interaction effects were estimated using the margins and margins plot commands in Stata. Paired and independent t-tests were conducted to compare within-group and between-group differences across PM_2.5_ seasons, respectively.

During the preparation of this manuscript, the authors used ChatGPT (OpenAI, GPT-4 model, 2025) to assist in editing and language refinement. The authors have carefully reviewed and edited the AI-generated content to ensure accuracy and take full responsibility for the final version of the manuscript.

## 3. Results

To assess the effects of metabolic health status, season, and their interaction on each biomarker, linear mixed-effects models were applied, adjusting for repeated measures within subjects. Statistically significant findings were further interpreted using predicted marginal means and 95% confidence intervals, with significance set at *p* < 0.05.

### 3.1. Baseline Characteristics of Participants

Table 1 presents the baseline characteristics of the 50 study participants, divided equally into healthy individuals and those with metabolic syndrome (MS). There was no statistically significant difference in gender distribution between the two groups, with females comprising 60.0% of the healthy group and 76.0% of the MS group (*p* = 0.225). The mean age was slightly higher in the MS group (52.2 ± 8.3 years) compared to the healthy group (48.3 ± 14.3 years), although the difference was not statistically significant (*p* = 0.177). Alcohol consumption patterns showed that 76.0% of healthy individuals and 52.0% of those with MS reported current alcohol use; however, this difference was not statistically significant (*p* = 0.140). The frequency of alcohol consumption over the past 30 days was similar between the groups.

Regarding smoking status, 72.0% of the healthy group and 80.0% of the MS group had never smoked, with no statistically significant difference being observed (*p* = 0.411). A small proportion of participants in both groups reported current or former smoking. The analysis of underlying diseases revealed that diabetes, hypertension, and hyperlipidemia were significantly more prevalent in the MS group compared to the healthy group. Diabetes, hypertension, and hyperlipidemia were present in 20.0%, 56.0%, and 28.0% of participants with MS, respectively. 

### 3.2. Seasonal Variation in PM_2.5_ Levels

PM_2.5_ data from the NTAQHI system showed clear seasonal differences across the five subdistricts of Samoeng District. During the high-exposure season (February–April 2023), the average PM_2.5_ concentration was approximately 67 µg/m^3^, while in the low-exposure season (May–July 2023), it dropped to around 7 µg/m^3^. These seasonal variations were consistent with local biomass burning patterns and have been reported in our previous study [22]. In the current analysis, we examine how these differences in environmental exposure relate to metabolic and hormonal parameters.

### 3.3. Hormonal and Metabolic Biomarker Trends

**Figure 3:**
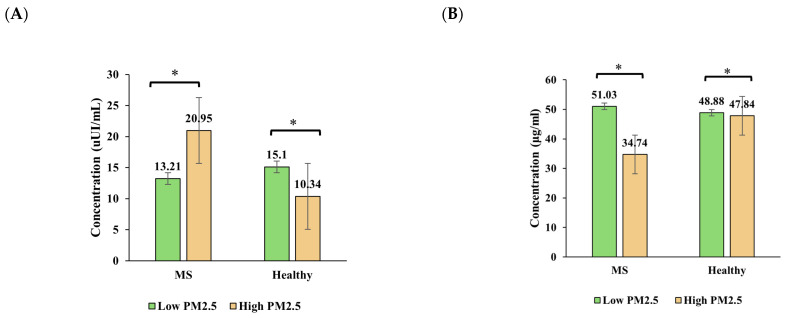

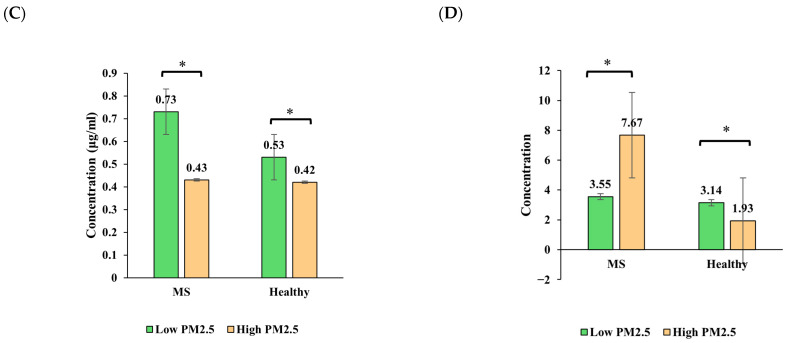
Seasonal variation in (**A**) insulin, (**B**) adiponectin, (**C**) leptin, and (**D**) HOMA-IR levels among healthy and metabolic syndrome (MS) groups. Bars show predicted marginal means ± 95% CIs from linear mixed-effects models. Asterisks indicate significant within-group seasonal differences (*p* < 0.05).

Figure 3 illustrates the seasonal variation in insulin, adiponectin, leptin, and HOMA-IR levels in healthy individuals and those with metabolic syndrome (MS) across the high- and low-PM_2.5_-exposure seasons.

Insulin levels significantly increased in the healthy group from the high-PM_2.5_ season to the low-PM_2.5_ season, rising from an estimated mean of 10.34 µIU/mL to 15.10 µIU/mL (*p* < 0.001). In contrast, the insulin levels in the MS group decreased from 20.95 µIU/mL to 13.21 µIU/mL (*p* < 0.001), indicating a differential seasonal response. A significant interaction effect (*p* = 0.020) was observed.

Adiponectin remained relatively stable in the healthy group across seasons, while a notable (but non-significant) increase was observed in the MS group from the high-PM_2.5_ season (34.74 µg/mL) to the low-PM_2.5_ season (51.03 µg/mL). The interaction effect was not significant (*p* = 0.192), but the group effect in the high-PM_2.5_ season was evident.

Leptin levels increased significantly in both groups across seasons, with a more pronounced rise in the MS group. In the high-PM_2.5_ season, leptin was similar between groups (~0.42–0.43 ng/mL), but in the low-PM_2.5_ season, the MS group reached 0.73 ng/mL, compared to 0.53 ng/mL in the healthy group. A strong group × season interaction was found (*p* < 0.001), suggesting that leptin is particularly sensitive to both metabolic status and seasonal environmental changes.

HOMA-IR increased modestly in healthy participants from 1.93 to 3.14, while the MS group showed a decrease from 7.67 to 3.55, although this was not statistically significant (*p* > 0.05). The interaction effect was not significant, but the main group effect was (*p* = 0.015), indicating overall higher insulin resistance in the MS group.

### 3.4. Statistical Comparisons Between Seasons and Groups

#### 3.4.1. Within-Group Seasonal Comparisons

Table 2 presents paired t-test results comparing hormonal and metabolic parameters within each group across the high- and low-PM_2.5_ seasons. In healthy individuals, leptin (*p* = 0.014) and HOMA-IR (*p* = 0.036) significantly increased during the low-exposure season, while insulin showed a near-significant rise (*p* = 0.051). In contrast, individuals with metabolic syndrome showed no statistically significant seasonal changes, although decreases in insulin and HOMA-IR were observed. Leptin significantly increased in the MS group as well (*p* < 0.001).

#### 3.4.2. Between-Group Comparisons by Season

The independent t-test results (Table 3) show that during the high-PM_2.5_ season, the MS group had significantly higher levels of insulin (*p* = 0.022) and lower adiponectin levels (*p* = 0.021) compared to healthy individuals. A marginal difference in leptin was also noted (*p* = 0.058). During the low-PM_2.5_ season, leptin levels were significantly higher in the MS group (*p* = 0.001), while the differences in insulin, adiponectin, and HOMA-IR were not statistically significant.

#### 3.4.3. Interaction Effects of Group and Season

The results from the two-way repeated-measures ANOVA (Table 4) indicated significant group × season interaction effects for both insulin (*p* = 0.020) and leptin (*p* < 0.001), suggesting differential seasonal responses between healthy participants and those with MS. A significant main effect of metabolic group was also observed for HOMA-IR (*p* = 0.015), indicating overall higher insulin resistance in the MS group across both seasons. No significant interaction or seasonal effects were detected for adiponectin.

## 4. Discussion

This study evaluated the seasonal variation in insulin, adiponectin, leptin, and HOMA-IR levels among healthy individuals and those with metabolic syndrome (MS) in relation to ambient PM_2.5_ exposure in Chiang Mai, Thailand. Our results demonstrated significant seasonal effects on metabolic and hormonal biomarkers, particularly for insulin and leptin, with pronounced group × season interaction effects. These findings support emerging evidence that fine particulate matter (PM_2.5_) acts as a metabolic disruptor and exerts differential physiological effects depending on individual health status [23].

Insulin levels were significantly higher in participants with MS during the high-PM_2.5_ season and declined significantly during the low-exposure season. Conversely, healthy individuals showed an increase in insulin concentrations across the same period. The significant interaction effect indicates that exposure to ambient air pollution may differentially influence insulin dynamics depending on an individual’s baseline metabolic health [24]. Chronic exposure to PM_2.5_ is known to impair glucose metabolism and promote insulin resistance through systemic oxidative stress, mitochondrial dysfunction, and low-grade inflammation [25]. PM_2.5_ may also alter pancreatic β-cell function and disrupt insulin signaling pathways, as observed in both human and animal studies [26]. A cohort study from China found similar seasonal insulin changes, linking elevated PM_2.5_ to increased insulin resistance, particularly among those with underlying metabolic disorders [27]. However, the observed rise in insulin levels among healthy individuals during the low-PM_2.5_ season may reflect compensatory physiological mechanisms rather than pollution-induced metabolic dysfunction [28]. This pattern could also be influenced by other unmeasured seasonal factors such as dietary intake, physical activity, or psychosocial stress, which were not captured in our study. Importantly, the lack of a parallel significant increase in HOMA-IR suggests preserved insulin sensitivity in this group [29].

The leptin levels increased significantly in both groups across seasons, with a stronger response being observed in the MS group. Leptin, a key adipokine regulating satiety and energy expenditure, is also a marker of adipose inflammation [30]. The observed seasonal rise in leptin may reflect pollution-induced alterations in adipose tissue homeostasis [31]. Elevated leptin concentrations in individuals with metabolic syndrome may further exacerbate leptin resistance, contributing to weight gain and impaired glucose metabolism [32]. Several epidemiological studies have reported associations between PM_2.5_ exposure and increased circulating leptin, especially among individuals with a higher BMI or metabolic dysfunction [33]. The strong interaction effect found in our study aligns with these observations and highlights leptin’s sensitivity to both environmental and metabolic influences. It is important to recognize that metabolic syndrome is a multifactorial condition influenced not only by environmental and lifestyle factors but also by genetic predisposition [34]. Several studies have identified variants in genes such as the fat mass and obesity-associated (FTO) gene and other loci related to insulin signaling and lipid metabolism as contributors to metabolic dysregulation [35]. These genetic factors may modulate individual susceptibility to environmental exposures, including air pollution. Therefore, the observed associations between PM_2.5_ exposure and metabolic or hormonal outcomes in our study may be partly shaped by underlying genetic variation that was not assessed. Our findings should be interpreted in this context, and future studies incorporating genotypic data are warranted to better understand the gene–environment interaction in metabolic syndrome pathogenesis.

Adiponectin levels remained stable in the healthy group but showed a non-significant upward trend in the MS group across seasons. Adiponectin is a protective adipokine that promotes insulin sensitivity and has anti-inflammatory properties. Its expression is typically reduced in individuals with obesity and metabolic syndrome [36]. Air pollution has been shown to suppress adiponectin levels via oxidative and inflammatory pathways, particularly in high-exposure environments [37]. While we did not observe statistically significant seasonal changes, the directionality of the effect aligns with previous studies, and the lack of significance may be due to sample size limitations.

HOMA-IR values increased significantly in healthy individuals during the low-PM_2.5_ season, while a non-significant decrease was observed in the MS group. This pattern may reflect underlying differences in metabolic regulation or external confounders such as diet or physical activity, which were not fully controlled in this study. Notably, the MS group had consistently higher HOMA-IR levels than the healthy group across both seasons, consistent with the established role of insulin resistance in metabolic syndrome [38]. We observed a significant main effect of metabolic group, but no interaction effect was observed, suggesting persistent group-level differences that were independent of seasonal variation.

Baseline group differences in chronic disease prevalence, including hypertension, diabetes, and hyperlipidemia, were also consistent with known features of metabolic syndrome and support the validity of our group categorization. These conditions are known to increase vulnerability to the adverse health effects of air pollution, including vascular dysfunction and cardiometabolic outcomes [39,40].

Our findings contribute to the growing literature identifying PM_2.5_ as an environmental risk factor for endocrine and metabolic disorders. Studies from North America, Europe, and Asia have consistently linked long-term exposure to PM_2.5_ with increased risk of type 2 diabetes, obesity, metabolic syndrome, and cardiovascular disease [41]. The mechanisms through which PM_2.5_ exerts these effects are complex and include systemic inflammation, epigenetic modification, dysbiosis of the gut microbiota, and disruption of the hypothalamic–pituitary–adrenal (HPA) axis [42]. The seasonal air pollution pattern in Chiang Mai, driven largely by agricultural biomass burning, offered a unique natural exposure model to examine the short-term metabolic effects of PM_2.5_. Importantly, the group × season interactions observed in insulin and leptin responses underscore that individuals with metabolic vulnerability may experience greater physiological disturbances in response to environmental stressors. This supports a precision public health approach to environmental risk mitigation.

This study offers several notable strengths. First, it employed a repeated-measures design, allowing each participant to serve as their own control across two distinct pollution seasons. This approach enhances the reliability of comparisons by minimizing between-subject variability. Second, the application of linear mixed-effects modeling allowed for robust analysis of both fixed and random effects, improving the statistical precision while accounting for within-subject correlations. Third, the inclusion of both healthy individuals and those with metabolic syndrome enabled subgroup analyses to explore how baseline metabolic health influences vulnerability to environmental exposures. Finally, the setting in Chiang Mai, which is characterized by well-defined seasonal PM_2.5_ variation due to biomass burning, provided a natural and relevant model to examine the real-world effects of air pollution on metabolic and hormonal parameters.

However, this study also has limitations. The relatively small sample size may have reduced the power to detect more subtle differences, especially for adiponectin and HOMA-IR. PM_2.5_ exposure was assessed using ambient air monitoring data from subdistrict-level sensors rather than individual-level measurements, which may have introduced exposure misclassification. Although PM_2.5_ was the primary pollutant of interest due to its dominance during seasonal haze events in Chiang Mai, we acknowledge that other co-existing pollutants (e.g., ozone, NO_2_, or volatile organic compounds) may also influence metabolic outcomes. Future studies should incorporate multi-pollutant exposure assessments to better disentangle the specific contributions of each pollutant. Additionally, other potentially confounding variables such as dietary intake, physical activity, psychosocial stress, and indoor air pollution were not controlled, which could influence metabolic outcomes. Lastly, the study population was limited to adults residing in a rural area, which may affect the generalizability of findings to other populations, including those living in urban settings or children.

Future studies should aim to incorporate detailed assessments of lifestyle factors (e.g., diet, activity, indoor exposures), genetic susceptibility, and individual-level exposure measurements to better understand the complex interactions between environmental and biological determinants of metabolic syndrome.

## 5. Conclusions

This study demonstrates that seasonal variation in PM_2.5_ exposure is associated with significant changes in insulin and leptin levels, with more pronounced responses among individuals with metabolic syndrome. These findings provide further evidence that air pollution may act as a metabolic stressor, contributing to hormonal dysregulation and increased risk of chronic disease. Efforts to reduce air pollution exposure, particularly during high-PM_2.5_ seasons, may be critical in protecting metabolic health, especially among high-risk populations.

## Figures and Tables

**Table 1:** Baseline characteristics of participants.

Characteristics	Healthy (*n* = 25)	Metabolic Syndrome (*n* = 25)	Total (*n* = 50)	*p*-Value
Gender, N (%)				0.225
Male	10 (40.0)	6 (24.0)	16 (32.0)	
Female	15 (60.0)	19 (76.0)	34 (68.0)	
Age (yr) mean ± SD	48.3 ± 14.3	52.2 ± 8.3	50.72 ± 11.8	0.177
Alcohol consumption N (%)				0.140
Never Drinks	6 (24.0)	12 (48.0)	18 (36.0)	
Drinks	19 (76.0)	13 (52.0)	32 (64.0)	
Frequency of Alcohol consumption over 30 days N (%)				
Every day	-	-	-	
Almost every day	-	-	-	
Every other day	3 (15.8)	3 (23.1)	6 (18.8)	
Every week	4 (21.1)	2 (15.4)	6 (18.8)	
Every month	9 (47.4)	6 (46.2)	15 (46.9)	
Do not drink	3 (15.8)	2 (15.4)	5 (15.6)	
Smoking N (%)				0.411
Never smokes	18 (72.0)	20 (80.0)	38 (76.0)	
Smokes	4 (16.0)	1 (4.0)	5 (10.0)	
Quit smoking	4 (12.0)	4 (16.0)	7 (14.0)	
Quit smoking (years) mean ± SD	21 ± 5.6	22.8 ± 16.1	22 ± 10.8	

**Table 2:** Comparison of hormonal and metabolic parameters between high- and low-PM_2.5_ seasons (within-group).

Parameter	Group	High PM_2.5_ (Mean ± SD)	Low PM_2.5_ (Mean ± SD)	*p*-Value (Paired *t*-Test)
Insulin (µIU/mL)	Healthy	9.3 ± 8.2	14.9 ± 12.1	0.051 *
	Metabolic Syndrome	22.0 ± 25.5	13.7 ± 8.0	0.214
Adiponectin (µg/mL)	Healthy	50.8 ± 28.9	49.2 ± 28.7	0.835
	Metabolic Syndrome	32.8 ± 22.8	50.4 ± 57.4	0.185
Leptin (ng/mL)	Healthy	0.39 ± 0.1	0.5 ± 0.2	0.014 *
	Metabolic Syndrome	0.44 ± 0.1	0.8 ± 0.2	0.000 *
HOMA-IR	Healthy	1.9 ± 1.7	3.1 ± 2.4	0.036 *
	Metabolic Syndrome	7.7 ± 16.6	3.6 ± 2.4	0.353

* *p*-values < 0.05, indicating statistically significant association.

**Table 3:** Comparison between healthy and metabolic syndrome groups in each season (between-group).

Parameter	Season	Healthy (Mean ± SD)	MS Group (Mean ± SD)	*p*-Value (Independent *t*-Test)
Insulin (µIU/mL)	High PM_2.5_	9.3 ± 8.2	22.0 ± 25.5	0.022 *
	Low PM_2.5_	14.9 ± 12.1	13.7 ± 8.0	0.714
Adiponectin (µg/mL)	High PM_2.5_	50.8 ± 28.9	32.8 ± 22.8	0.021 *
	Low PM_2.5_	49.2 ± 28.7	50.4 ± 57.4	0.924
Leptin (ng/mL)	High PM_2.5_	0.39 ± 0.1	0.44 ± 0.1	0.058
	Low PM_2.5_	0.5 ± 0.2	0.8 ± 0.2	0.001 *
HOMA-IR	High PM_2.5_	1.9 ± 1.7	7.7 ± 16.6	0.089
	Low PM_2.5_	3.1 ± 2.4	3.6 ± 2.4	0.494

* *p*-values < 0.05, indicating statistically significant association.

**Table 4:** Two-way repeated-measures ANOVA—interaction effect of group and season. * Statistically significant at *p* < 0.05.

Parameter	Factor	Z	*p*-Value	95%CI
Insulin	Metabolic group	2.65	0.008 *	2.8–18.5
	Season	1.47	0.143	−1.6–11.1
	Metabolic group × Season interaction	−2.34	0.020 *	−23.0–2.0
Adiponectin	Metabolic group	−1.47	0.140	−30.51–4.3
	Season	0.15	0.884	−12.9–15.0
	Metabolic group × Season interaction	1.31	0.192	−7.6–38.1
Leptin	Metabolic group	0.26	0.795	−0.07–0.09
	Season	3.9	0.000 *	0.06–0.17
	Metabolic group × Season interaction	3.9	0.000 *	0.1–0.3
HOMA-IR	Metabolic group	2.43	0.015 *	1.1–10.4
	Season	0.55	0.581	−3.1–5.5
	Metabolic group × Season interaction	−1.53	0.127	−12.2–1.5

## Data Availability

The data presented in this study are available upon request from the corresponding author.

## Abbreviations

The following abbreviations are used in this manuscript:

ELISA	Enzyme-linked immunosorbent assay
HDL-C	High-density lipoprotein cholesterol
HOMA-IR	Homeostatic Model Assessment of Insulin Resistance
HPA	Hypothalamic–pituitary–adrenal
MS	Metabolic syndrome
NTAQHI	Northern Thailand Air Quality Health Index
PM_2.5_	Fine particulate matter
RIHES	Research Institute for Health Sciences
